# Strong frequency dependence of vibrational relaxation in bulk and surface water reveals sub-picosecond structural heterogeneity

**DOI:** 10.1038/ncomms9384

**Published:** 2015-09-18

**Authors:** Sietse T. van der Post, Cho-Shuen Hsieh, Masanari Okuno, Yuki Nagata, Huib J. Bakker, Mischa Bonn, Johannes Hunger

**Affiliations:** 1 FOM Institute AMOLF, Science Park 104, 1098 XG Amsterdam, The Netherlands; 2 Max-Planck Institute for Polymer Research, Ackermannweg 10, 55128 Mainz, Germany

## Abstract

Because of strong hydrogen bonding in liquid water, intermolecular interactions between water molecules are highly delocalized. Previous two-dimensional infrared spectroscopy experiments have indicated that this delocalization smears out the structural heterogeneity of neat H_2_O. Here we report on a systematic investigation of the ultrafast vibrational relaxation of bulk and interfacial water using time-resolved infrared and sum-frequency generation spectroscopies. These experiments reveal a remarkably strong dependence of the vibrational relaxation time on the frequency of the OH stretching vibration of liquid water in the bulk and at the air/water interface. For bulk water, the vibrational relaxation time increases continuously from 250 to 550 fs when the frequency is increased from 3,100 to 3,700 cm^−1^. For hydrogen-bonded water at the air/water interface, the frequency dependence is even stronger. These results directly demonstrate that liquid water possesses substantial structural heterogeneity, both in the bulk and at the surface.

## Abstract (web summary)

The structural heterogeneity in liquid water is commonly believed to disappear beyond 50 fs due to the strong intermolecular interaction. Here, the authors show frequency-dependent vibrational relaxation, which indicates the persistence of structural heterogeneity on a picosecond timescale.

## Graphical abstract

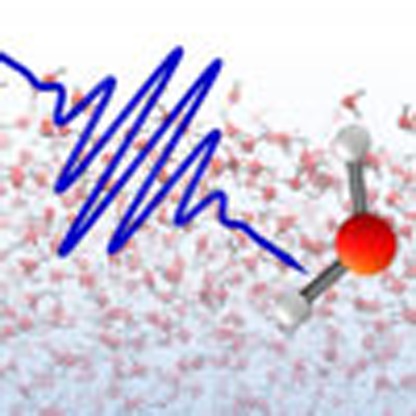

Water exhibits unique properties, distinctively different from other liquids, such as a high heat capacity, a high surface tension and a reduced density of the solid compared with the liquid. These properties are caused by the strong intermolecular interactions between water molecules, through the so-called hydrogen bond (H-bond) network^1^. Despite the relatively strong H-bond interaction in liquid water, the three-dimensional structure is very dynamic and H-bond breaking and reformation occurs on (sub-) picosecond timescales^2^. Although there is evidence from scattering experiments that liquid water exhibits substantial structural heterogeneity^3^, the quantum nature of the H-bond, in particular for light water, results in significant delocalization of the intermolecular interaction^4^,^5^, which may smear out the structural heterogeneity. Experimentally, information on the local intermolecular interaction is readily accessible using the OH stretching vibration of water^6^. For neat H_2_O, the infrared absorption band of the OH stretching vibration is remarkably broad^1^, much broader than the OH stretching band of isolated HOD molecules in D_2_O, where the vibrational modes are localized^7^. Similar broad vibrational bands are observed for interfacial water at the air/water interface^8^, for which the vibrational response can be selectively determined using infrared—visible (VIS) sum-frequency generation (SFG) spectroscopy^9^,^10^. It has, however, remained elusive whether the extremely broad bandwidth of the OH stretching band is a sole result of the delocalized nature^11^ and thus originates from strong intra- and intermolecular coupling^12^ or that the delocalization occurs on a smaller length scale than the structural heterogeneity, which implies that the OH stretching band is inhomogeneously broadened.

Because of the strongly coupled and delocalized nature of the OH oscillators in pure H_2_O, no appreciable heterogeneity has been reported for the OH stretch vibrational dynamics in bulk^12^,^13^,^14^ or at the air/water interface^15^. In particular, no discernible frequency dependence of the vibrational relaxation of the OH stretching vibration has been observed in ultrafast infrared experiments^13^,^14^,^15^,^16^,^17^,^18^,^19^,^20^ despite molecular dynamics simulations predicting a pronounced correlation of the vibrational lifetime with the number of water molecules in its first coordination shell^21^. Accordingly, the experimentally measured modulation of the OH oscillator frequency (spectral diffusion) has been reported to be very fast in bulk H_2_O (∼50–150 fs (refs 12, 13, 14)). Apparently, the frequency memory of an excited OH oscillator is extremely short, resulting in very fast and frequency-independent vibrational relaxation time for neat H_2_O (refs 13, 14). Thus, besides some very early Raman experiments^22^, which were due to limited temporal resolution controversially discussed^23^, there is no indication of any structural heterogeneity in neat H_2_O from state-of-the-art infrared experiments. In contrast, the vibrational lifetime of diluted OH oscillators (HOD in D_2_O), for which delocalization is reduced, shows a pronounced variation with frequency^24^ as a result of preferential transfer of the excess vibrational energy to the overtone of HOD's bending vibration^25^,^26^,^27^: OH groups with a vibrational frequency close to the bend overtone relax relatively quickly. Similar to isotopically diluted water, a reduction of the intermolecular coupling and consequently spectral diffusion is observed at the air/water interface^28^,^29^, as the density of water molecules is reduced (the number of water molecules in the air phase is negligible).

Here, we report on the vibrational energy relaxation (VER) of the OH stretching mode in bulk water and at the air/water interface. By probing the dynamics over a wide frequency range, including the far-red and far-blue edges of the vibrational absorption band, we elucidate an evidently continuous structural heterogeneity of neat water.

## Results

### Vibrational spectra of H_2_O

**Figure 1:**
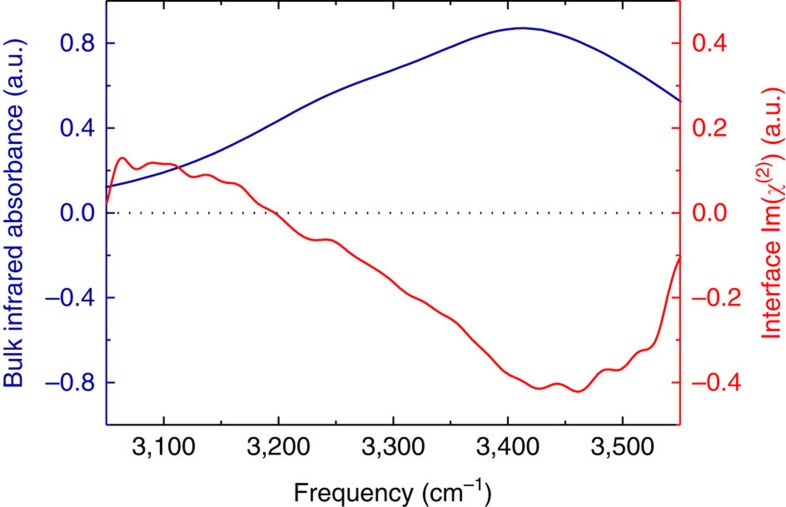
OH stretching band of H_2_O. The infrared absorption spectrum of bulk water and the Im[χ^(2)^] spectrum of the air/water interface in the hydrogen-bonded OH stretch region.

In bulk water, the vibrational infrared band of the OH stretching mode is centred at 3,400 cm^−1^ (Fig. 1) with a linewidth of several hundreds of wavenumbers. The vibrational response of interfacial water can be readily accessed with SFG spectroscopy. The SFG intensity is determined by the second-order susceptibility χ^(2)^, and the imaginary part of the second-order susceptibility, Im[χ^(2)^], can be determined using phase-resolved methods^30^,^31^. Im[χ^(2)^] constitutes the surface equivalent of the bulk infrared absorption spectrum. Moreover, a positive (negative) band in the Im[χ^(2)^] spectrum indicates the net up (down)-orientation of the OH stretch transition dipole moment at the air/water interface. The spectral shape of the Im[χ^(2)^] spectrum of water at the air/water interface is similarly broad as the bulk infrared absorption spectrum of H_2_O (Fig. 1)^30^. As detailed below, we monitor the vibrational dynamics after excitation with a narrowband infrared pump pulse in bulk using infrared pump-probe spectroscopy and at the air/H_2_O interface using interfacial infrared pump-SFG probe experiments by probing the vibrational response.

### Bulk water infrared pump-probe results

To study VER in bulk H_2_O, we use a narrow-band infrared excitation pulse to excite a subset of H_2_O molecules of a given frequency. We probe the temporal evolution of the excitation, which results in a modulation of the vibrational spectrum, with a second weak infrared probe pulse that measures the difference spectrum between the excited and non-excited sample, Δ*α*_iso_. The presence of an excited state population causes a reduction of the absorbance at the fundamental frequency (0→1 transition) of the oscillator (a negative feature in the transient spectra, Δ*α*_iso_<0) and an induced absorbance at somewhat red-shifted frequencies because of the excited state absorption (Δ*α*_iso_>0, 1→2 transition). The red-shift of the excited state absorption with respect to the fundamental frequency is a direct consequence of the anharmonicity of the OH oscillator. We note that after VER, persistent non-zero signals contribute to the transient spectra at the fundamental frequency, which originate from a local temperature rise because of the dissipation of the vibrational energy. This increase in temperature gives rise to a blue-shift of the OH stretching band because of a weakening of all H-bonds in water. To circumvent complications due to these thermal effects in determining the lifetime, we probe the excited state absorption at *ω*_probe_=2,900 cm^−1^, where the contribution of these thermal effects is small and thus can be easily corrected for (see Methods and Supplementary Figs 1–3 for details).

**Figure 2:**
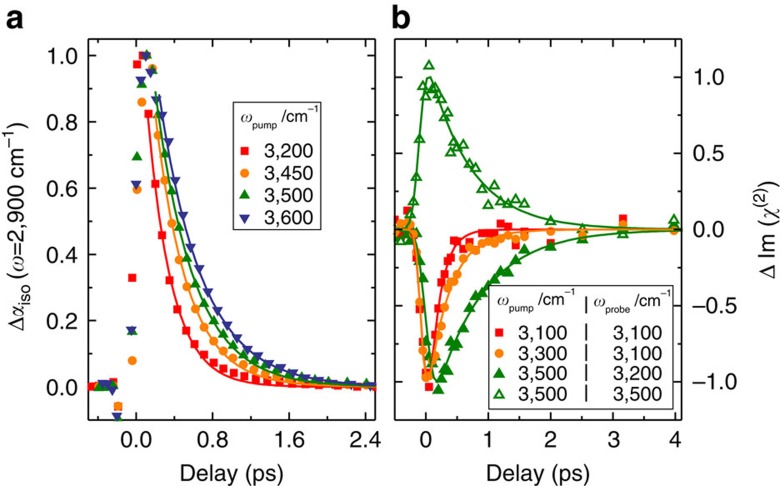
Vibrational dynamics at OH stretching frequencies. (**a**) Normalized infrared pump/probe data for H-bonded OH groups in bulk H_2_O. The delay traces are taken at *ω*_probe_=2,900 cm^−1^ with the pump frequencies centred at *ω*_pump_=3,200, 3,450, 3,500 and 3,600 cm^−1^. The data are corrected for the contribution of the thermalized transient spectrum (see Methods section for details). At 2,900 cm^−1^, the magnitude of this correction is >0.2 (see Supplementary Figs 1–3 for details). (**b**) Dynamics of the interfacial water molecules obtained using an infrared pump/HD-SFG probe scheme. The delay traces show data with the pump pulses centred at *ω*_pump_=3,500, 3,300 and 3,100 cm^−1^. The SFG probe frequency is set to spectral ranges where the contribution of thermalization to the signal is negligible.

**Figure 3:**
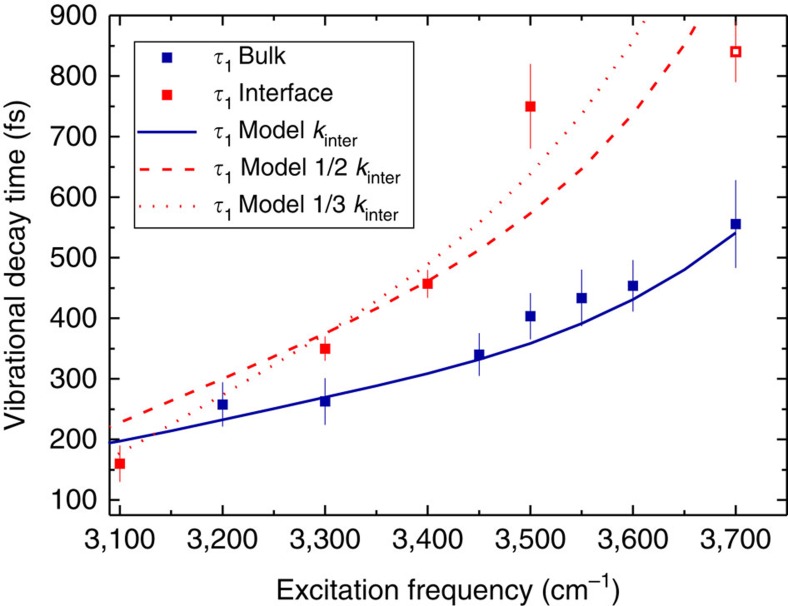
Vibrational relaxation time constants of bulk and interfacial H_2_O. Experimentally observed relaxation times *τ*_1_ of the OH stretch vibration of bulk (blue symbols) and interfacial (red symbols) H_2_O as a function of the OH stretch excitation frequency. The open-red symbol corresponds to the vibrational relaxation time of the free (non hydrogen-bonded) OH groups. Error bars for the infrared pump-probe decay times correspond to a 100% increase in the sum of the squared deviations of the fit of the kinetic model to the data shown in Supplementary Figs 2 and 3. Error bars for the infrared pump-SFG-probe decay times correspond to the standard error obtained using a Levenberg–Marquardt fit of a single exponential decay to the experimental data in Fig. 2b. The solid blue curve represents the *τ*_1_ time calculated using the model with the overtone of the bending vibration centred at 3,250 cm^−1^. The dashed (dotted) red line shows the *τ*_1_ time calculated using the same model with the bending overtone of interfacial water centred at 3,190 cm^−1^ and a twofold (threefold) reduced spectral diffusion rate (for details, see text and Methods section).

To study the frequency-dependent VER, we tune the excitation pulses to seven different frequencies, *ω*_pump_. The pump-probe traces are fit with a kinetic model in order to correct for the thermal contribution (that is, the time-dependent contribution of the ‘hot ground state' to the transient spectra resulting from sample heating is subtracted, see Methods for details). Typical normalized infrared pump/probe traces for bulk H_2_O, which are corrected for the thermalization of the sample, are shown in Fig. 2a. It is apparent from these data that the vibrational relaxation is significantly slowed-down with increasing *ω*_pump_. This slow-down is directly observable in the raw data as the thermal contribution to the transient spectra is small (see Supplementary Fig. 1 for details). We extract the decay time of the transient signals, *τ*_1_, by fitting a single exponential decay to the transient signals shown in Fig. 2a. The variation of *τ*_1_ with *ω*_pump_ is summarized in Fig. 3. From these measurements, we find *τ*_1_ to increase from ∼250 fs for *ω*_pump_<3,300 cm^−1^ to *τ*_1_∼550 fs for *ω*_pump_=3,700 cm^−1^. Remarkably, the value of *τ*_1_ of (560±70) fs for *ω*_pump_=3,700 cm^−1^ is close to the value observed for the OH vibration of HOD molecules in D_2_O (740 fs)^32^,^33^. We note that the vibrational relaxation for *ω*_pump_=3,500 cm^−1^ is somewhat slower compared with what has been reported previously^23^. However, this early study^23^ used broader excitation pulses, and as a consequence the reported relaxation time represented a weighted average over a wider frequency range (∼200 cm^−1^). Thus, this report is fully consistent with our present findings. It is also important to point out that the measured transient signals with tunable *ω*_pump_ and fixed *ω*_probe_=2,900 cm^−1^ contain both contributions from VER and from spectral diffusion as the absorbance of the excited state absorption at this frequency depends on the frequency of the excited oscillator.

The slow-down of the decay of the transient signals measured at 2,900 cm^−1^ with increasing pump frequency shows that full spectral equilibration is not achieved within the timescale of the experiment (∼1 ps). Our results show that vibrational relaxation of neat liquid water varies with frequency, with substantially slower population relaxation for OH stretch oscillators centred at the blue-side of the OH absorption band. Although our experiments are conceptually similar to previous two-dimensional (2D) infrared experiments, the frequency dispersion of *τ*_1_ has not been recognized before^12^,^13^,^14^. This apparent contradiction can be explained by noting that the dispersion of *τ*_1_ is most pronounced at blue-shifted pump-frequency where the transition dipoles of OH oscillators are small^34^. As a consequence, the slowly decaying signals at high frequencies are easily overwhelmed by the more intense transient signals at the centre of the OH stretching band and can be easily overlooked in commonly used 2D infrared plots. As demonstrated in the analysis presented below, the present results are fully consistent with previous 2D infrared experiments^12^,^13^,^14^.

### Interfacial infrared pump-SFG probe experiments

In Fig. 2b, we show the corresponding experiments for the air/H_2_O interface. In these infrared pump/heterodyne-detected SFG (HD-SFG) probe experiments, we excite a subset of OH oscillators with a narrowband infrared pump pulse centred at *ω*_pump_=3,100, 3,300, 3,400 and 3,500 cm^−1^. Subsequently, we detect the modulation of the vibrational response of the interfacial water molecules using a HD-SFG detection scheme^29^,^35^,^36^. Here, we detect the modulation of the imaginary part of the second-order response, ΔIm[*χ*^(2)^], which is the surface analogue of the transient absorption spectrum of bulk water (see Methods for details). For the data shown in Fig. 2b, we select the SFG detection frequency (such as *ω*_pump_/*ω*_probe_=3,100 cm^−1^/3,100 cm^−1^, 3,300 cm^−1^/3,100 cm^−1^, 3,500 cm^−1^/3,200 cm^−1^ and 3,500 cm^−1^/3,500 cm^−1^), so that the contribution of the thermalization is zero (that is, the contribution of the heated ground state to the transient spectra at long delay times is negligible)^29^. Thus, the ΔIm[*χ*^(2)^] data in Fig. 2b directly correspond to the dynamics of the excited state population. The signals recorded at *ω*_probe_=3,100 cm^−1^ (Fig. 2b) directly reveal, also at the interface, a pronounced frequency dependence of the vibrational relaxation dynamics: the transient signals decay with a time constant of *τ*_1_=(350±20) fs and (160±30) fs for excitation at 3,300 and 3,100 cm^−1^, respectively. For the excitation at 3,500 cm^−1^, the transient signal decays substantially slower. Note that for these data, our experimental frequency range allows probing the population relaxation of the same type of O–H oscillator both at its fundamental 0→1 transition frequency of 3,500 cm^−1^ and at its excited absorption 1→2 transition frequency of 3,200 cm^−1^. At both frequencies, the contribution of the hot ground state is negligible. The relaxation times (*τ*_1_=(700±50) fs for *ω*_probe_=3,500 cm^−1^ versus *τ*_1_=(750±70) fs for *ω*_probe_=3,200 cm^−1^) at these two probe frequencies are identical within their respective errors, demonstrating the robustness of the experimental approach. At 3,700 cm^−1^, which is the resonance frequency of the (non H-bonded) free OH groups, the decay has a time constant of (840±50) fs (refs 37, 38). The population decay times of interfacial water molecules at different excitation frequencies are summarized in Fig. 3. From this figure it is clear that the frequency dependence of the relaxation time is even more pronounced at the air/H_2_O interface compared with bulk H_2_O.

From the measured relaxation times *τ*_1_ for both bulk and surface (Fig. 3), it is clear that there is a remarkably strong dispersion in the relaxation times despite the strong intra- and intermolecular coupling of the OH vibrations of liquid H_2_O. The observation of strongly dispersive relaxation times directly shows the structural heterogeneity of water, both in the bulk and at the interface: On a timescale of ∼1 ps, a variety of differently H-bonded water molecules persist, each with its own distinct relaxation time. We note that this conclusion follows directly from the data, independent of the model presented below.

### Modelling dispersive vibrational relaxation

The observed time constants *τ*_1_ are spectrally averaged values of the intrinsic vibrational population relaxation time constants *T*_1_, and the dispersion in *T*_1_ will therefore be even stronger than that of *τ*_1_. The value of *τ*_1_ at a particular observation frequency is determined by both the frequency dispersion of the intrinsic relaxation time constants *T*_1_ and the rate of spectral equilibration. To quantitatively describe the experimentally observed dispersion in the relaxation times *τ*_1_, we use a kinetic model that accounts for spectral diffusion and vibrational relaxation. In this model, we decompose the experimental infrared absorption spectrum of bulk H_2_O into individual Lorentzian oscillators with a linewidth of *Γ*=150 cm^−1^ (evenly spaced by 2 cm^−1^ over frequencies ranging from 3,000 to 3,800 cm^−1^). The choice for this value of the linewidth will be discussed below. The frequency-dependent transition dipole moment for each Lorentzian was taken from the literature^34^.

Spectral diffusion in liquid water can originate from distinctively different molecular processes: anharmonic couplings of the OH stretching vibration to lower frequency modes and structural fluctuations of the hydrogen-bonded water structure strongly affect the resonance frequency of an OH oscillator^5^,^14^,^39^. In addition, dipole–dipole coupling results in a transfer of the excitation population to neighbouring OH oscillators^19^,^21^,^40^, which can have slightly different resonance frequencies. Despite the different possible molecular origins of the spectral diffusion process, they all have in common that the modulation rate must slow down with increasing amplitude of the frequency fluctuations: large frequency excursions take more time. For convenience, we thus model spectral diffusion in a phenomenological manner by taking the transfer rate from one Lorentzian oscillator to another with different frequency to be proportional to the spectral overlap integral of both oscillators and proportional to the relative number density of the accepting mode in water multiplied by an intrinsic transfer rate *k*_inter_. We adjusted the rate constant *k*_inter_ of the transfer between OH oscillators to reproduce the spectral dynamics of liquid H_2_O (ref. 12) (see Supplementary Discussion 1). The parameters *k*_inter_ and the natural linewidth of the Lorentzian oscillator *Γ* are interdependent; we here set *Γ*=150 cm^−1^, but note that a range of combinations of *k*_inter_ and *Γ* can reproduce the experimental observations.

As detailed above, our data provide direct evidence for a dispersion of the intrinsic vibrational relaxation time *T*_1_. This dispersion is likely the result of the energy mismatch between the relaxing OH stretch vibration and the combination of modes that accept the vibrational energy. There are several potential combinations of intra- and intermolecular modes that can accept the energy of the excited OH stretch vibration. However, there is substantial evidence that the overtone of the bending vibration forms the major pathway for VER^21^,^26^,^27^,^41^. Hence, we assume the vibrational relaxation of the OH stretching vibration to occur via energy transfer to the bending overtone. The overtone of the H_2_O bending vibration is described as a Gaussian band centred at 3,250 cm^−1^ with a linewidth of 125 cm^−1^ (ref. 41). We take the intrinsic population relaxation time *T*_1_ (that is, the loss of vibrational excitation) to be proportional to the spectral overlap of each Lorentzian OH oscillator (with a linewidth *Γ*=150 cm^−1^) with the Gaussian spectrum of the overtone of the H_2_O bending vibration multiplied with an intrinsic relaxation rate *k*_bend_ (ref. 18). This approach is quite general: the spectral profiles represent the effects of fluctuations (independent of the origin of the fluctuations) on the energy levels that are needed to compensate the energy mismatch. With increasing central frequency of the Lorentzian OH oscillator, the spectral overlap with the overtone of the bending mode decreases and *T*_1_ becomes longer.

Starting with an initial Gaussian excitation profile (centred at *ω*_pump_ with a linewidth of *σ*=100 cm^−1^), we simulate the temporal evolution of the excitation population numerically with a time step of 0.2 fs. The total population of all excited oscillators as a function of time is then convolved with a 70 fs (full-width half-maximum (FWHM)) instrument response function and fit to a single exponential decay. The resulting time constants for the exponential decay are compared with our experimental results (see Supplementary Fig. 4 for details). Details of the modelling can be found in the Methods section below.

As can be seen in Fig. 3, by only adjusting the intrinsic rate for energy relaxation to the overtone of the bending overtone, *k*_bend_, and the intrinsic spectral diffusion rate, *k*_inter_, we can accurately reproduce the observed variation of the relaxation time constant *τ*_1_ with frequency (blue solid line in Fig. 3) in bulk H_2_O. The relaxation to the bend overtone directly yields the frequency dependence of the intrinsic *T*_1_ time constant. Within our model, the intrinsic *T*_1_ time constant varies from ∼100 fs at 3,100 cm^−1^ to ∼1.5 ps at 3,600 cm^−1^ in a strongly nonlinear manner. This frequency dependence is determined, in the model, by the spectral characteristics of the OH stretch and bend overtone, and the associated values for *k*_inter_ and *k*_bend_. A similar nonlinear frequency dependence of the intrinsic *T*_1_ could have been obtained with a more complex model involving several relaxation channels with different energy gaps. However, such a description is not required to account for the experimental observations.

Despite being rather phenomenological, our model is also in broad accordance with the results of previous 2D infrared experiments: from the modelled time-dependent excited state populations we can readily extract 2D infrared spectra (see Supplementary Discussion 1 and Supplementary Fig. 5 for details). The time-dependent centre line slope, which is commonly used as a measure for the excitation frequency memory in 2D infrared experiments, extracted from our model decays with a time constant of ∼140 fs, which agrees well with the previously reported decays of the centre line slope of bulk H_2_O (50–180 fs)^12^,^13^,^14^ (see Supplementary Fig. 6 for details). In turn, this means that the commonly used 2D infrared plots are not very sensitive to dispersive vibrational dynamics at the edges (that is, at the blue edge for the OH stretching band of H_2_O) of the vibrational bands. Our model is further in broad agreement with state-of-the-art *ab-initio* molecular dynamics simulations that predict the first spectral moment of the transient spectra to equilibrate with a ∼140-fs time constant after excitation at 3,600 cm^−1^ (ref. 5), whereas our model predicts a ∼180-fs equilibration (see Supplementary Discussion 2 and Supplementary Fig. 7 for details).

We now proceed with modelling the results at the interface. For interfacial water molecules, the bending vibration has been reported to be red-shifted, with respect to bulk water^42^. Hence, in modelling the infrared pump/HD-SFG probe experiments, we assume the overtone of the bending vibration to be located at 3,190 cm^−1^. It has further been observed that spectral diffusion of interfacial water is slower than in bulk water^28^,^29^. Several factors influence the spectral diffusion rate at the interface: At the interface, the density of water is reduced by a factor of two, the number of water molecules in the gas phase being negligible. Hence, the reduced number of oscillators that can accept the vibrational energy is expected to reduce the spectral diffusion rate by a factor of 2 (refs 28, 29). Moreover, molecular dynamics simulations have indicated that the spectral dynamics at the interface is slowed-down by even more than a factor of 2 in comparison to bulk water, as the H-bond switching, which also contributes to the spectral equilibration, is three times slower at the interface compared with the bulk^43^. In Fig. 3, we show the effect of a twofold and the effect of a threefold reduction of the spectral diffusion rate (reduced *k*_inter_) on the value of *τ*_1_ as obtained from our model with all other parameters (except the position of the bending overtone) kept the same as for bulk water. As can be seen from the dashed red line in Fig. 3, a twofold reduction of the spectral diffusion rate leads to an enhanced dispersion of *τ*_1_. Reducing *k*_inter_ by a factor of 3 quantitatively describes the interfacial effects on the vibrational population relaxation, as measured in our infrared pump/HD-SFG probe experiments for excitation frequencies ranging from 3,100 to 3,500 cm^−1^. We note that comparison between the model and data is inappropriate for *ω*_pump_=3,700 cm^−1^, because the experiment probes, at this frequency, the outermost non-H-bonded OH oscillators, pointing towards the air phase^38^. These OH groups are rather isolated because of the lack of H-bonding and have been demonstrated to have very different coupling to other H_2_O molecules compared with H-bonded OH groups (ref. 38), which is not accounted for in our model.

In conclusion, we report a strong frequency dependence of the vibrational relaxation of the OH stretch vibrations of water, both in the bulk and at its surface. For bulk water, the vibrational relaxation time shows a smooth increase from ∼250 fs at the red side of the OH stretching band to ∼550 fs at the blue edge of the infrared absorption band. A similar increase by a factor of two from the red side to the blue side has been reported for the OH vibration of HOD dissolved in D_2_O (ref. 24), showing that also for HDO, spectral diffusion from the red and blue edges of the OH stretch band is sufficiently slow for the heterogeneity in H-bond strengths to be expressed in variations of the vibrational lifetime. Despite the very fast spectral diffusion and accelerated vibrational relaxation of the OH stretch vibration in pure H_2_O, we show that this intrinsic heterogeneity persists in H_2_O. For interfacial H_2_O, the variation of the vibrational decay time is even more pronounced than for bulk water, with *τ*_1_ increasing from ∼150 fs at 3,100 cm^−1^ to ∼750 fs at 3,500 cm^−1^.

We show that the experimental results can be quantitatively described with a model that includes spectral diffusion and intramolecular VER to the overtone of the bending vibration. We thus conclude that the longer vibrational lifetime for blue-shifted OH oscillators (compared with OH oscillators at the red side of the OH stretching band) results from a reduced frequency overlap with the bending overtone of H_2_O. The larger dispersion of *τ*_1_ at the interface compared with bulk water can be well explained from the slower spectral equilibration at the interface. Thus, our results demonstrate that in water the coupling between OH oscillators absorbing at extreme (high or low) frequencies and the majority of the water molecules is quite weak and the dispersion of the vibrational relaxation evidences the continuous intrinsic structural heterogeneity of water. Our findings also imply that this structural heterogeneity of water results in strong inhomogeneous broadening of the OH stretch absorption band of H_2_O both in the bulk and at the interface. Taking the results from recent molecular dynamics simulations into account that find a pronounced correlation between the vibrational relaxation time and the local coordination number of water molecules correlation^21^, our results also imply that the band shape of the OH stretching band of aqueous solutions can be used to infer the distribution of the local H-bonded structure of the water molecules. Specifically, the VER rate reflects the local H-bonding network, with faster relaxation occurring for more strongly and/or highly coordinated OH groups, and slower relaxation for less/more weakly coordinated water molecules. Finally, our finding also has implications for the dissipation of excess energy released by chemical reactions: the isolated and slowly relaxing nature of the water OH groups at high frequencies imply that not all water molecules are capable of rapidly transferring the excess energy. Hence, even in the absence of specific interactions of solutes with water, continuum hydration models may fail as such models do not account for the heterogeneous structure and energy dissipation characteristics of water.

## Methods

### Pump probe experiments

To study the vibrational dynamics of the OH stretching vibration of neat H_2_O, both in bulk and at the interface, we use ultrafast laser spectroscopies. In these experiments, an intense femtosecond infrared laser pulse is used to vibrationally excite a subset of OH oscillators. The vibrational excitation leads to distinct changes in the vibrational spectra. To study the frequency-dependent spectral dynamics, the bandwidth of the excitation pulse is much narrower than the very broad vibrational spectrum of the OH stretching vibration (see Fig. 1 for details). The spectral changes, typically recorded as transient spectra (difference spectra between excited and non-excited samples), are temporally monitored as a function of frequency. For the infrared pump-probe experiments, a weak infrared probe pulse is used to interrogate the vibrational response. In the infrared pump/HD-SFG-probe experiment, which probes due to its selection rules (forbidden in centrosymmetric media) specifically interfacial water molecules, an infrared and a VIS pulse are overlapped. The generated sum-frequency provides vibrational spectra of interfacial water.

### Bulk infrared pump-probe experiments

The infrared pump-probe experiments are based on a regenerative Ti:Sapphire amplified laser system (Coherent), providing pulses at 800 nm with a duration of 35 fs and a pulse energy of 3.5 mJ at a repetition rate of 1 kHz. The output of the laser is split into three portions: 850 μJ are used to pump a homebuilt optical parametric amplifier (OPA)^44^. The signal and idler pulses of the OPA are mixed in a silver-gallium-disulfide crystal (thickness 1.2 mm) to generate the difference frequency. This yields mid-infrared probe pulses with a centre frequency of 3,000 cm^−1^, a pulse energy of 5 μJ and a FWHM of 300 cm^−1^. A wedged ZnSe window is used to generate a probe and a reference pulse from the output of the OPA, with the reflection of the front side being used as the probe pulse and the back-reflection used as a reference pulse. Another 850 μJ of the 800 nm pulses are used to pump a commercial parametric amplifier (TOPAS, Light Conversion). The idler pulses of the TOPAS with a wavelength of ∼2,200 nm are frequency doubled in a *β*-Bariumborate crystal. The residual ∼1,100 nm pulses are used as a seed pulse in another parametric amplification process in a Potassium titanyl phosphate crystal (thickness 10 mm), which is pumped by 1 mJ of 800 nm pulses. The resulting infrared pump pulses have a pulse energy of 16 μJ and a spectral bandwidth of ∼50 cm^−1^ FWHM. A translational stage is used to control the timing of the infrared pump-pulses. The pump is transmitted through a *λ*/2 plate to rotate its polarization at 45° with respect to the probe pulse polarization. A mechanical chopper placed in the pump path is used to block every second pump pulse, to allow for active background subtraction.

The pump, probe and reference pulses are focused in the sample using an off-axis parabolic mirror, with the pump and the probe pulse spatially overlapping. A mechanically rotated polarizer after the sample allows selecting the polarization components of the probe pulse parallel or perpendicular to the pump polarization. After recollimation of the beams with a second parabolic mirror, the probe and the reference pulses are spectrally dispersed with an imaging spectrograph and detected on two separate lines of a 3 × 32 mercury-cadmium-telluride (Infrared Associates) detector array. The measurement of the reference allows for a frequency-resolved correction for shot-to-shot fluctuations of the probe-pulse energy. The pump-induced absorption change, 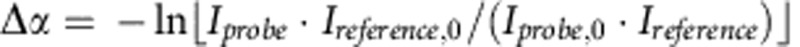, is detected via the transmitted intensities, *I*, on the detector, where subscript ‘0' refers to the infrared intensities recorded without pump excitation. The absorption changes parallel, Δ*α*_*II*_(*ω*,*t*), and perpendicular, Δ*α*_⊥_(*ω*,*t*), to the pump pulse excitation are used to construct the isotropic transient signals, Δ*α*_*iso*_(*ω*,*t*)=[Δ*α*_*II*_(*ω*,*t*)+2Δ*α*_⊥_(*ω*,*t*)]/3, which represents the vibrational population dynamics and are independent of orientational processes. The temporal evolution of the transient signal at 2,900 cm^−1^ for different pump frequencies is shown in Supplementary Fig. 1.

Following previously described procedures, we fit a relaxation model to the experimental transient spectra^45^,^46^. In this model, the excited state population decays with a time constant *τ*_1_ to an intermediate state, which is transiently populated. In the intermediate state, the energy is not yet fully equilibrated over the system. The full thermal equilibration of the system occurs with a relaxation time τ*, resulting in the fully thermalized state (heated ground state). The value of τ* was fixed to 550 fs, which is the mean value of the equilibration time obtained from a free fit to all measurements. The excited state and the equilibrated thermalized state have each their associated transient spectra, whereas the transient spectrum of the intermediate state is assumed to be zero. All transient spectra were analysed at probing frequencies ranging from 2,800 to 3,000 cm^−1^, at which the transient signals are dominated by the excited state absorption. The contributions of the individual states to the fit can be seen in Supplementary Figs 2 and 3. For the data shown in Fig. 2a, the contribution of the hot ground state has been subtracted (based on the time-dependent population of this state as exemplarily shown in Supplementary Figs 2 and 3).

### Infrared pump/HD-SFG probe spectroscopy

The experimental setup has been described in detail elsewhere^29^. In brief, a Ti:Sapphire regenerative amplifier (Spitfire Ace, Spectra-Physics) was used to generate laser pulses (centre wavelength: 800 nm, bandwidth: FWHM 30 nm, pulse width: 40 fs). The amplifier produces ∼5 mJ of energy/pulse at a repetition rate of 1 kHz. Two commercial OPAs (TOPAS-C, Spectra-Physics) are both pumped with 1 mJ of the 800 nm pulses. The signal and idler pulses from one TOPAS-C were used in a difference frequency mixing process in a silver gallium disulfide (AgGaS_2_) crystal, resulting in 3 μJ infrared pulses at a central wavelength of ∼3,030 nm (∼3,300 cm^−1^) with a FWHM of 300 cm^−1^, which was used as infrared probe. For the experiments with infrared pump at 3,100 and 3,300 cm^−1^, the probe wavelength was tuned by rotating the AgGaS_2_ crystal to be centred at 3,200 cm^−1^. The infrared pump pulses were generated analogous to the infrared pump-probe experiments yielding ∼100 μJ mid-infrared pulses with a FWHM of 100 cm^−1^. Another fraction of the 800 nm pulses was spectrally narrowed by an etalon (SLS Optics Ltd.) to 9 cm^−1^ and was used for the VIS probe. The infrared pump/HD-SFG probe measurements were performed in reflection geometry. The VIS and infrared probes were first focused onto a gold mirror to generate a SFG signal, which is used as a local oscillator (LO). The VIS, infrared probes and LO were refocused onto the sample by a concave mirror. The LO passed through a fused silica plate (1 mm thickness) to be delayed in time before reflected by the concave mirror. The VIS probe, infrared probe, LO and infrared pump beams are all in the same plane with incident angles of the VIS, infrared probe and infrared pump of 40°, 51° and 31° with respect to the surface normal, respectively. The SFG from the sample and LO were guided into a spectrograph and interfered with each other in the frequency domain. The resultant interference fringe pattern combined with the reference spectrum from a z-cut quartz allows obtaining complex spectra of the second-order nonlinear susceptibility χ^(2)^. The energy of the infrared pump, infrared probe and VIS probe at the sample were 40, 1 and 4 μJ per pulse, respectively. The sample was distilled Millipore water (18 MΩ-cm resistivity). The samples were placed in a home-built Teflon trough, which was rotated at 7 r.p.m. to avoid cumulative heating. The infrared pump/HD-SFG probe spectra were recorded under p/ssp (infrared pump/SFG, VIS probe, infrared probe) polarization. The infrared pump pulse was variably delayed with respect to the SFG probe signal using a mechanical delay line. The differential infrared pump/HD-SFG probe was computed as the difference between the integrated intensities with and without the pump with an integrated bandwidth of 100 cm^−1^. The interference spectrum, *I*(*ω*) obtained from the spectrometer is expressed as the squared total field intensity 
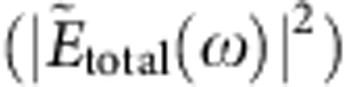
:


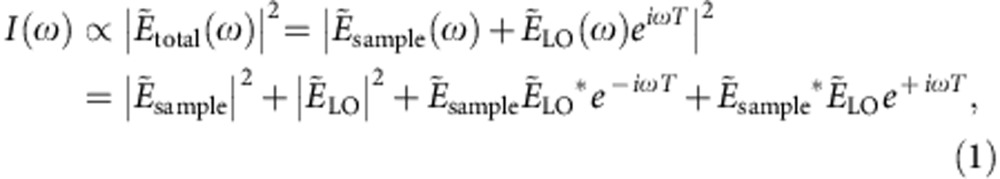


where 
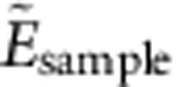
 and 
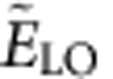
 are the SFG electric fields from the sample and LO, respectively. *T* is the time delay between the signals from the sample and the LO. The spectrum is converted to time-domain via inverse-Fourier-transformation. After filtering out the first, second and the fourth terms, the third term was extracted and Fourier transformation was applied to obtain the complex spectrum. The same procedure is applied to quartz as a reference. By dividing the complex spectrum of the sample and the quartz, the HD-SFG signal (Im[χ^(2)^]) is obtained. The differential signal (ΔIm[χ^(2)^]) was computed by subtracting the pumped signal with the unpumped signal and normalized to the maximum of the bleach of the signal.

### Modelling vibrational relaxation dynamics

To model our experimental results, we assume the infrared absorption band of the OH stretching vibration in neat H_2_O to be composed of individual Lorentzian oscillators centred at a frequency *ω*_0_


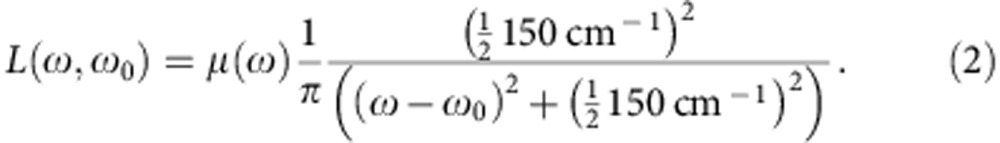


The frequency-dependent transition dipole, *μ*(*ω*), was taken from the literature^34^. Note that the value of *μ*(*ω*) is not well established at high frequencies, thus, we assume *μ*(*ω*) to be constant above 3,707 cm^−1^.

The distribution of oscillators in liquid water is calculated from the infrared absorption spectrum of water, *α*(*ω*):


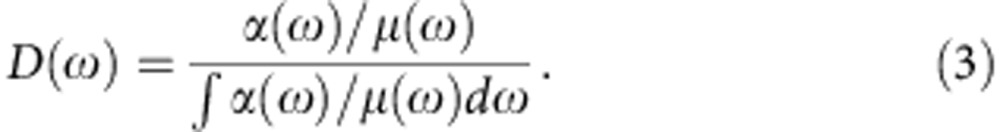


To account for spectral diffusion, the transfer rate of the vibrational energy from an oscillator A centred at frequency *ω*_A_ to an oscillator B centred at frequency *ω*_B_ is given by the number density of acceptor modes times the spectral overlap of oscillators A and B:


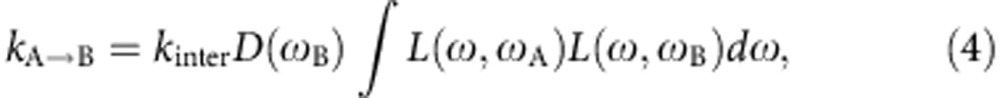


where *k*_inter_ is the intrinsic transfer rate.

In a similar manner, the intramolecular VER rate from an oscillator A to the overtone of the H_2_O bending vibration is given by:


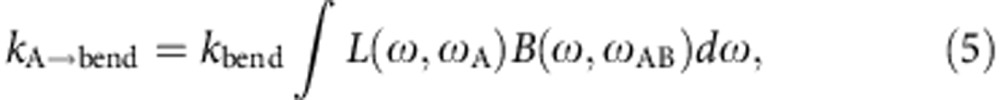


with *k*_bend_ being the intrinsic vibrational relaxation rate. Note that 1/*k*_A→bend_ corresponds to the intrinsic vibrational relaxation rate *T*_1_ as discussed above. The band shape of the overtone of the bending vibration is assumed to have a Gaussian line shape:


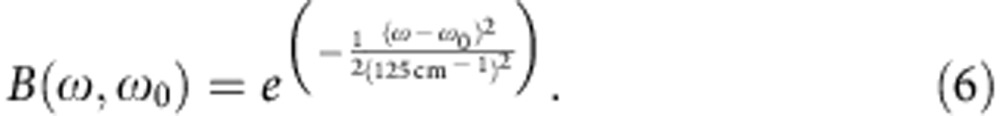


At time zero, the frequency-dependent initial population was obtained using a Gaussian excitation profile:


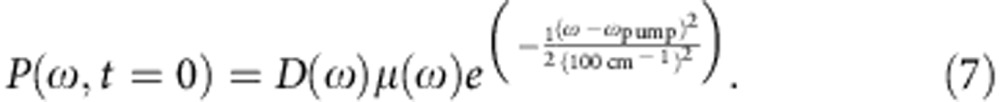


The temporal evolution of the frequency-dependent population was then calculated numerically at intervals of 0.2 fs using the rates *k*_A→B_ for transfer between different oscillators and *k*_A→bend_ for dissipation of vibrational energy to the bending vibration. For numerical simulation, a total number of 400 equally spaced Lorentzian oscillators were used, spanning frequencies ranging from 3,000 to 3,800 cm^−1^.

The absorbance of the total excitation population as a function of time is given by:


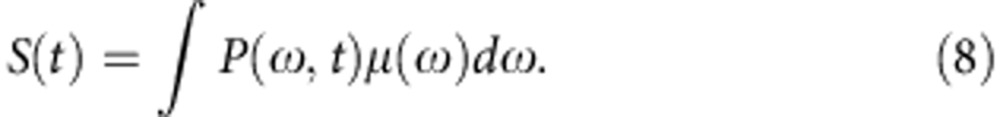


The thus obtained decay of *S*(*t*) for different excitation frequencies for bulk water was convolved with a Gaussian (70 fs FWHM) instrument response function. Typical *S*(*t*) decays as extracted from the model are shown as dashed lines in Supplementary Fig. 4 for different values of *ω*_pump_. We fit a single exponential decay with a decay time of *τ*_1_ to the modelled *S*(*t*) values. Such fits are shown as solid lines in Supplementary Fig. 4. The decay times extracted from these fits are shown as lines in Fig. 3.

## Additional information

**How to cite this article:** van der Post, S. T. *et al.* Strong frequency dependence of vibrational relaxation in bulk and surface water reveals sub-picosecond structural heterogeneity. *Nat. Commun.* 6:8384 doi: 10.1038/ncomms9384 (2015).

## Supplementary Material

Supplementary Information Supplementary Figures 1-7, Supplementary Discussion and Supplementary References
