# Supplementary material for: Strong frequency dependence of vibrational relaxation in bulk and surface water reveals sub-picosecond structural heterogeneity
Source: Nat Commun. 2015 Sep 18;6:8384. doi: 10.1038/ncomms9384 (PMC4595750; doi:10.1038/ncomms9384)
Supplement: Supplementary Information — Supplementary Figures 1-7, Supplementary Discussion and Supplementary References [file ncomms9384-s1.pdf]

Supplementary Figures

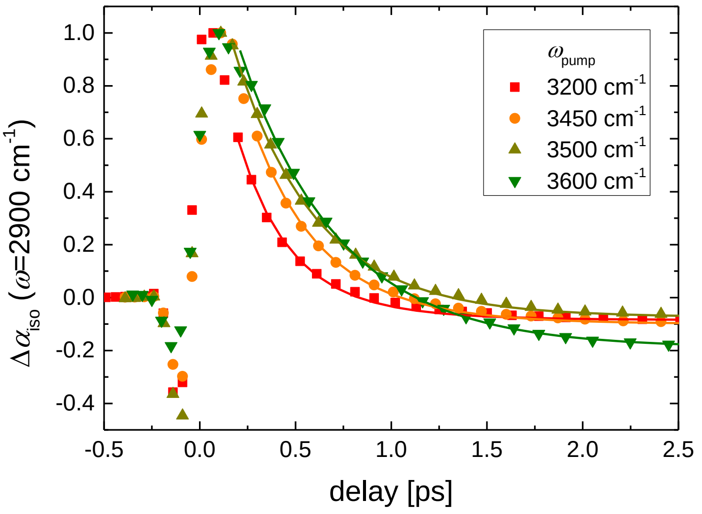

Supplementary Figure 1: Raw infrared pump-probe traces. Transient signals of the infrared pump-probe experiments as function of delay time at ω_{probe} = 2900 cm^{-1} for different excitation frequencies. Symbols show experimental data and the solid lines show fits of the relaxation model (see Methods section of the main manuscript) to the data.

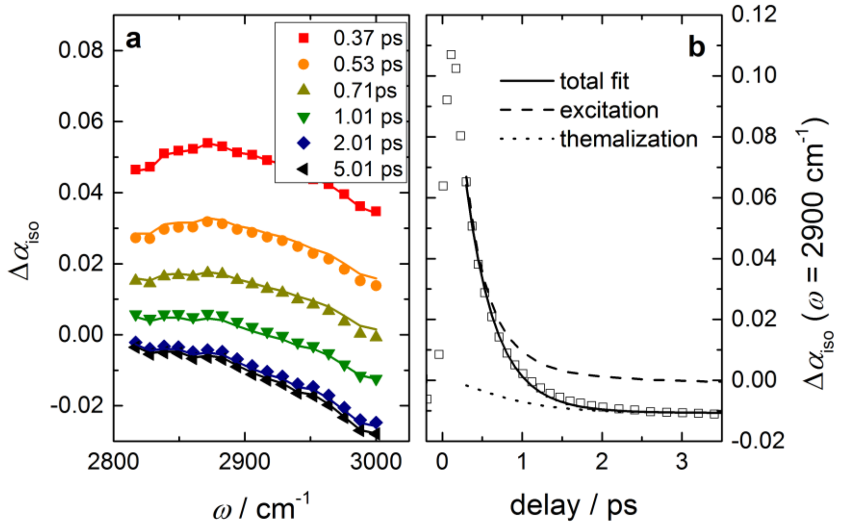

Supplementary Figure 2: Fit of the IR pump-probe data with 3450 cm^{-1} excitation. (a) Transient isotropic spectra, $\Delta\alpha_{iso}$, at different delay times with ω_{pump} = 3450 cm^{-1}. (b) Transient signals at ω_{probe} = 2900 cm^{-1} with ω_{pump} = 3450 cm^{-1}. Symbols are experimental data of the infrared pump-probe experiments and the solid lines correspond to fits using the kinetic model (see Methods section of the main manuscript). The dashed and dotted lines indicate the contribution of the excited state absorption and the thermal signal, respectively, to the total fit.

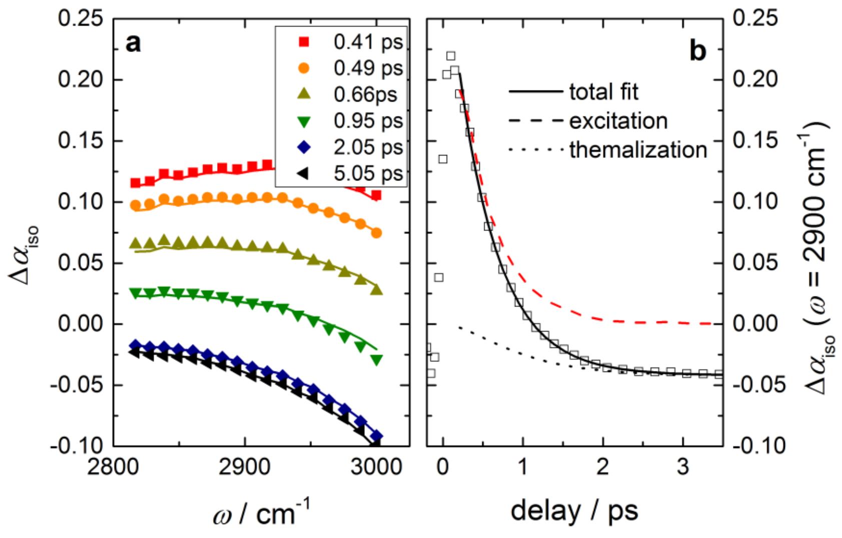

Supplementary Figure 3: Fit of the IR pump-probe data with 3600 cm^{-1} excitation. (a) Transient isotropic spectra, $\Delta\alpha_{iso}$, at different delay times with ω_{pump} = 3600 cm^{-1}. (b) Transient signals at ω_{probe} = 2900 cm^{-1} with ω_{pump} = 3600 cm^{-1}. Symbols are experimental data of the infrared pump-probe experiment and the solid lines correspond to fits using the kinetic model (see Methods section of the main manuscript). The dashed and dotted lines indicate the contribution of the excited state absorption and the thermal signal, respectively, to the total fit.

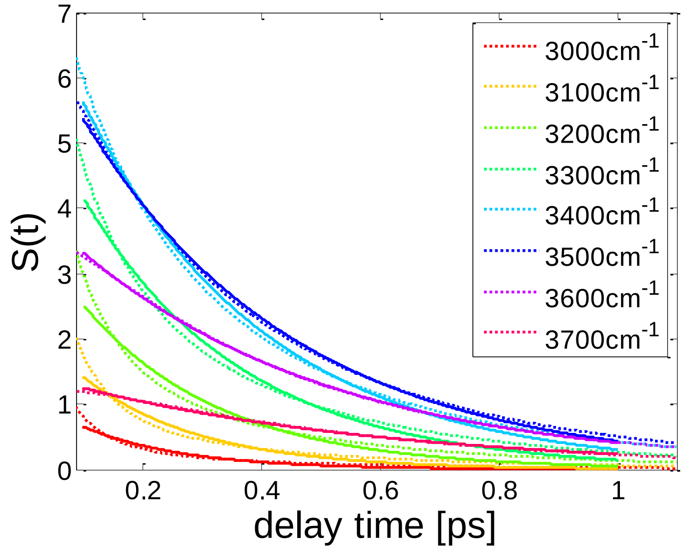

Supplementary Figure 4: Modelled excitation populations. Simulated total absorbance of the excited state population for different pump frequencies. Dashed lines show the modelled values of $S(t)$, while the solid lines show single exponential fits (see Methods section of the main manuscript).

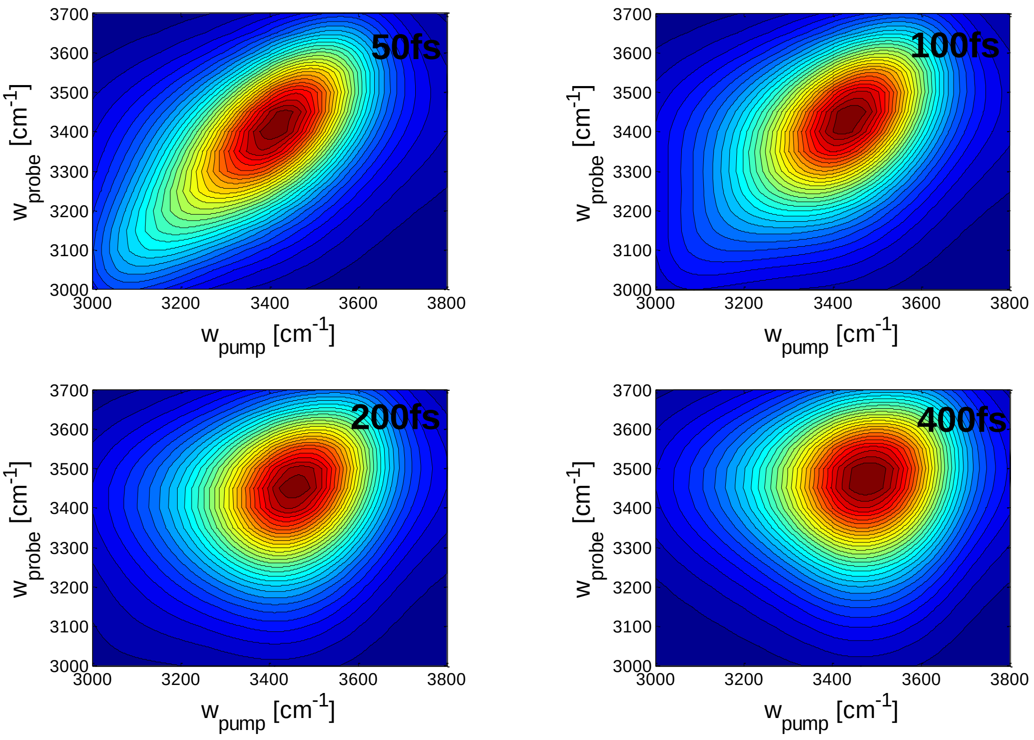

Supplementary Figure 5: Modelled two-dimensional IR spectra. 2D-IR spectra extracted from modelling the temporal evolution of the frequency dependent excited state population for delay times values of 50, 100, 200, and 400 fs after excitation.

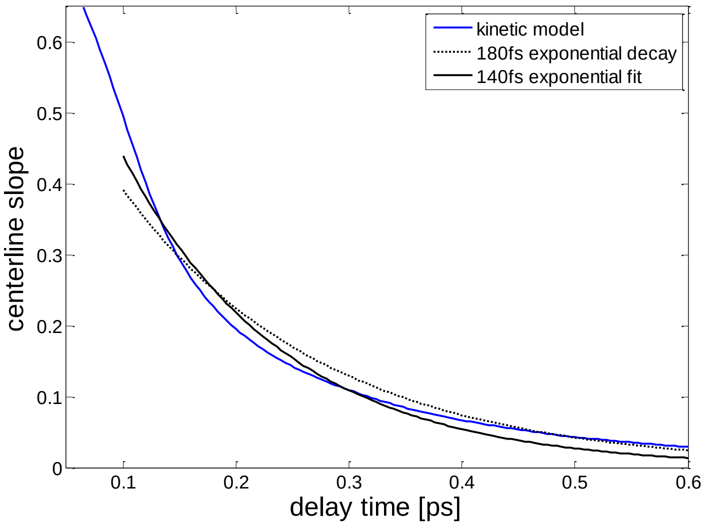

Supplementary Figure 6: Modelled center line slopes of the 2D-IR spectra. Center line slope as a function of delay time, as extracted from our modelled 2D-IR spectra (solid blue line). The modelled centerline slopes were convolved with a 70 fs (FWHM) instrument response function. The dashed lines shows a single exponential decay with a time constant of 180 fs, which has been reported based on experimental data.[1] A single exponential fit of the modelled decay (solid black line) gives a 140 fs decay time.

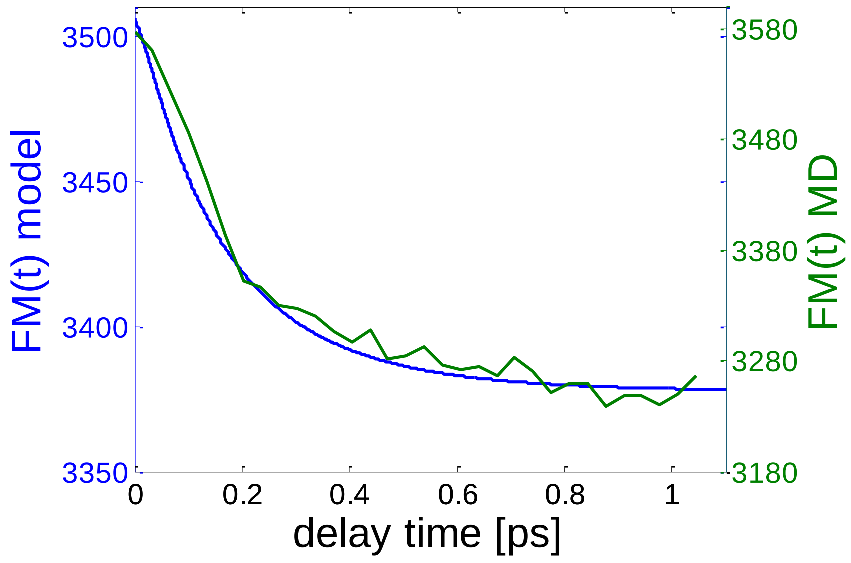

Supplementary Figure 7: Time dependent first spectral moment. First spectral moment of the excited state population after excitation at 3600 cm^{-1}. The solid blue line (left axis) shows the results from our model (see Supplementary Discussion 1). The green solid line (right axis) represent results that have been obtained using ab initio molecular dynamics simulations.[2]

Supplementary Discussion

1. Comparison of the modelled vibrational dynamics to results from two-dimensional infrared spectroscopy.

As indicated in the main manuscript, our model gives rise to 2D-IR spectra, which are in broad concordance with experimental results. Modelled 2D-IR spectra for different delay times are shown exemplarily in Supplementary Figure 5. In analogy to the analysis of experimentally measured 2D-IR spectra,[1] we extract the decay of the centerline slope of the modelled 2D-IR spectra, which is a measure for the loss of frequency-frequency correlation (i.e. spectral diffusion). As can be seen in Supplementary Figure 6, the thus extracted decay is in reasonable agreement with experimental results.[1]

2. Comparison of the modelled vibrational dynamics to results from *ab initio* molecular dynamics simulations.

We also show the equilibration of the first spectral moment after excitation at 3600 cm^{-1} in Supplementary Figure 7 and compare it to state of the art *ab initio* molecular dynamics simulations.[2] The first spectral moment was extracted from the population-weighted average frequency of the excited state (see Methods Section of the main manuscript):

$$FM(t) = \frac{\int P(\omega,t)\omega d\omega}{\int P(\omega,t) d\omega}$$

In order to isolate the contribution of spectral diffusion from the vibrational dynamics, we do not allow for vibrational relaxation (k_{bend}=0) for this comparison. The spectral diffusion dynamics of this model is compared with the recent *ab initio* molecular dynamics simulation data at the BLYP/DZVP level of theory, which is displayed in Supplementary Figure 7. This shows that the temporal evolution of $FM(t)$ as extracted from our model is in excellent agreement with the computational results. We note that the *ab initio* simulations somewhat underestimate vibrational frequencies due to the excessively strong anharmonicity of the BLYP functionals.[2,3] Thus, while the dynamics agree, the absolute values of $FM(t)$ from to our model are somewhat larger than predicted by the *ab initio* MD simulations.

Supplementary References

1. Ramasesha, K., De Marco, L., Mandal, A. & Tokmakoff, A. Water vibrations have strongly mixed intra- and intermolecular character. *Nat. Chem.* **5,** 935–40 (2013).

2. Nagata, Y., Yoshimune, S., Hsieh, C.-S., Hunger, J. & Bonn, M. Ultrafast Vibrational Dynamics of Water Disentangled by Reverse Nonequilibrium Ab Initio Molecular Dynamics Simulations. *Phys. Rev. X* **5,** 021002 (2015).

3. Lee, H.-S. & Tuckerman, M. E. Dynamical properties of liquid water from ab initio molecular dynamics performed in the complete basis set limit. *J. Chem. Phys.* **126,** 164501 (2007).
